# The application of a surgical face mask over different oxygen delivery devices; a crossover study of measured end-tidal oxygen concentrations

**DOI:** 10.1186/s12871-022-01602-y

**Published:** 2022-03-07

**Authors:** Kate Brown-Beresford, John Currie, Venkatesan Thiruvenkatarajan

**Affiliations:** 1 Southern Adelaide Local Health Network, Adelaide, SA Australia; 2 Department of Anaesthesia, The Queen Elizabeth Hospital, Central Adelaide Local Health Network, Adelaide, SA Australia; 3 Department of Anaesthesia, Noarlunga Health Service, Adelaide, SA Australia; 4 Department of Anaesthesia, Flinders Medical Centre, Adelaide, SA Australia; 5 Discipline of Acute Care Medicine, University of Adelaide, Adelaide, SA Australia

## Abstract

**Background:**

The application of a surgical face mask over oxygen delivery devices is now a widespread recommendation in the setting of the Coronavirus disease pandemic. This addition is designed to reduce droplet spread, but this also changes the nature of these devices, and may alter the amount of oxygen delivered to a patient. This research investigated how placing a surgical face mask over both a simple plastic mask (“Hudson mask”) and nasal cannula altered the concentration of available oxygen measured at the nares.

**Methods:**

We measured the inspired and end-tidal oxygen concentrations of five healthy non-smoking volunteers. Oxygen was delivered via nasal cannula and also a simple plastic face mask, at flow rates of 2, 4, 6 and 8 l per minute, with and without an overlying surgical face mask.

**Results:**

Adding a surgical mask over nasal cannula caused an appreciable rise in the end-tidal oxygen concentrations at all the measured oxygen flow rates 2, 4, 6, 8 L/minute. With the Hudson mask, there was a rise in oxygen concentration at 4 and 6 L/minute. For example, at a flow rate of 4 l/min via nasal cannula, available oxygen concentration increased from 24 to 36%, and via the Hudson mask the concentration rose from 27 to 38%.

**Conclusions:**

The addition of a surgical face mask over both nasal cannula and a Hudson mask resulted in an increased available oxygen concentration. This may be valuable where more advanced oxygen devices are not available, or alternatively providing adequate supplemental oxygen at lower flow rates and thus making critical savings in oxygen usage.

**Supplementary Information:**

The online version contains supplementary material available at 10.1186/s12871-022-01602-y.

## Background

In the setting of the Coronavirus disease (COVID-19) pandemic, there have been numerous changes to day-to-day clinical practice in order to reduce infection transmission in hospitals. One of these changes is the recommended use of surgical face masks on patients with either suspected or proven respiratory infections [1–3]. The rationale is simple; these masks significantly reduce the spread of infectious droplets and subsequent viral transmission [3–9]. Known or suspected patients with COVID-19 often have significant respiratory symptoms and frequently require oxygen therapy [10]. Oxygen therapy further raises concerns of aerosolisation of viral particles from the nasopharynx of patients, and an even greater spread to the surrounding environment [2, 9, 11]. This further supports the use of overlying surgical masks in these patients and it is now a common sight to see a patient receiving oxygen therapy with a surgical facemask applied over the top [1–3]. The Australasian College of Emergency Medicine has now incorporated this practice into their resuscitation algorithm [12].

There is a strong literature base which details the amount of oxygen delivered via both nasal cannula and the simple plastic mask (often referred to as a Hudson mask) at different gas flow rates and breathing patterns [13–19]. To date however, there is no published literature that evaluates the changes in delivered oxygen concentration by the addition of an overlying face mask to either of these devices. This is relevant in the setting of the COVID-19 pandemic and into the future, given the increasing recommendations for the application of surgical masks over these devices for infection control.

The aim of this research project was to measure the change in the concentration of oxygen that is delivered to a healthy individual once a surgical mask is worn over a simple oxygen delivery system.

## Methods

### Study design

This was a simple crossover study and ethical approval was granted by the South Adelaide Local Health Network (SAHLN) Office for Research under the low and negligible risk research pathway OFR No. 122.20.

Oxygen (O_2_) concentrations were measured in each subject with and without a surgical mask in place. Blinding of the subjects was not able to be performed, as the subjects could easily detect which O_2_ device they were wearing. Randomisation of the various set ups was not done, as we felt that bias in the simple measurements taken would be unlikely, and this allowed a streamlined and uniform procedure protocol for each subject.

O_2_ concentrations were measured by attachment of a gas sampler just outside the nares on the face, as seen in Fig. 4 (Appendix). Oxygen was then delivered first via nasal cannula only, at O_2_ flow rates of 2, 4, 6 and 8 l per minute (L/min), and then again with the application of an overlying surgical mask. The process was then repeated for the Hudson mask readings, as depicted in Fig. 5 (Appendix). For each of these variables, measurements of fraction of inspired oxygen (FIO_2_) and end-tidal oxygen concentration (ETO_2_) were recorded at 30 s intervals for a period of 5-min at each O_2_ delivery rate. This provided 11 individual sampling measurements per oxygen delivery flow rate for each of the experimental conditions, per subject. At least a 3-min interval was provided in-between the increase in rate and commencement of measurements, and also between different devices to allow an equilibrium of gases to establish, and avoid any carry over effect.

### Participants

Five healthy non-smokers (three women and two men) volunteered to take part in this study, as per the inclusion and exclusion criteria outlined in Table 1. This was an “initial” sample number, chosen to obtain baseline values. We had the intention to recruit further volunteers if measurements varied widely between individuals. At the time of our project, supplies were low in our facility, and daily inventories of PPE were being made, and we were mindful of the non-therapeutic nature of our study. Fortunately, wide variation did not occur between subjects, and more subjects would not have added any meaningful additional value to the study.

**Table 1:** Inclusion and Exclusion criteria for participant recruitment

Inclusion Criteria	Exclusion Criteria
• ASA Physical Status Classification System grade 1 – healthy patient [20] • Non-smoker • Age > 18 • Voluntary subject	• Pre-existing lung disease • Current or ex-smoker • Any current infective or respiratory symptoms • Any other significant medical condition requiring treatment with regular medication

Subjects breathed normally during the study period with occasional speaking in an attempt to be more congruous with a real patient scenario. In addition, a resuscitation mannequin was also fitted with the sampling line in an identical fashion, to obtain values without the effects of any respiration.

### Oxygen devices

The oxygen delivery devices used were a Hudson RCI Nasal Cannula with 2.1 m Star Lumen Tubing and a Hudson RCI Medium Concentration Elongated SEE-THRU Oxygen Mask [21]. The surgical masks used were Medline Surgical Fluid-Resistant Face Mask With Ties Level 3 [22].

The oxygen delivery device conditions studied were:

1. Nasal cannula only (*n* = 5)
2. Nasal cannula + overlying surgical mask (*n* = 5)
3. Hudson mask only (*n* = 5)
4. Hudson mask + overlying surgical mask (*n* = 5)

### Gas sampling

Gas was sampled from the area immediately adjacent to the subjects’ nares using an anaesthetic machine gas sampling line through an in-linePALL Medical Pall PharmAssure 25 mm Hydrophobic Vent Filter, as demonstrated in Fig. 4 (Appendix) [23]. The anaesthetic machine used was the GE Healthcare Aisys CS^2^with the integrated Carescape monitor [24]. The sampling flow is 200 ml per minute and the oxygen analyser in this system is of the paramagnetic type.

### Measurements

The readings from the GE Aisys CS2 are in a dual format; FIO_2_ and ETO_2_. The oxygen sampled concurrent with the peak value of carbon dioxide (CO_2_) is assigned by the machine to be the ETO_2_ concentration, and the oxygen concentration sampled concurrent with the lowest CO_2_ concentration is assigned to be the FIO_2_. Both values were recorded separately. With the mannequin setup, where no CO_2_ is measurable, all recorded values of ETO_2_ and FIO_2_ were identical.

### Data analysis

In a study by Waldau, Larson and Bonde, the ETO_2_ reading provided by the machine was described to be a more consistent data point, compared to the displayed FIO_2_ values [13]. We concur with this (see discussion, below, for full argument) and therefore, the ETO_2_ values were used for determination of the primary outcome measures of this study.

ETO_2_ and FIO_2_ data were subsequently grouped by oxygen delivery system and O_2_ flow rates. The mean of each group was then established with the accompanying calculation of standard deviation and standard error.

## Results

### Nasal cannula

**Fig. 1:**
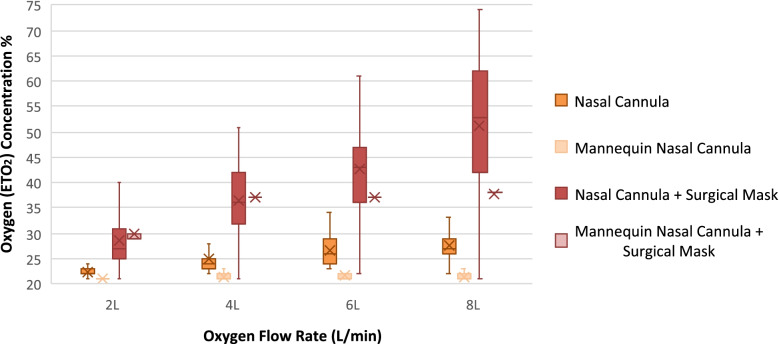
Box and whisker plot comparison of all measured ETO_2_ concentrations using nasal cannula and different mask strategies

The addition of a surgical facemask to nasal cannula increased the mean ETO_2_ values sequentially by 6, 12, 16 and 24%, at respective O_2_ flow rates of 2, 4, 6 and 8 L/min (absolute change), as depicted below in Fig. 1. With the non-respiring mannequin, a similar increase in mean ETO_2_ concentration was observed once a face mask was placed, however increasing the flows of oxygen appeared to only offer an additional benefit when flow rates increased from 2 to 4 L/min, after which it then reached a plateau, as demonstrated below in Fig. 1.

### Simple oxygen mask (Hudson mask)

**Fig. 2:**
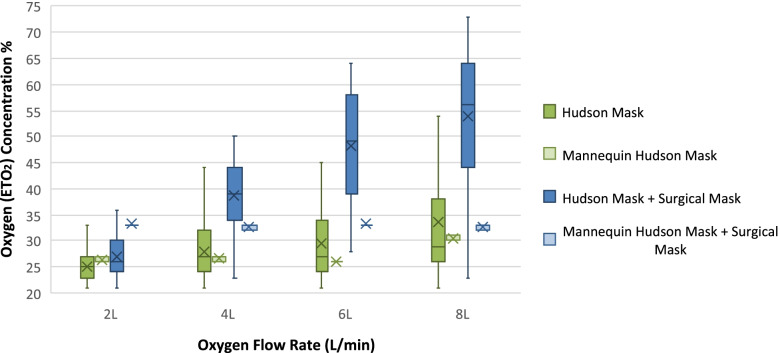
Box and whisker plot comparison of all measured ETO_2_ concentrations using a Hudson mask, with and without an overlying surgical mask

Placement of a surgical facemask over a Hudson mask also increased the mean ETO_2_ values sequentially by 2, 11, 18 and 20% at respective O_2_ flow rates of 2, 4, 6 and 8 L/min (absolute change). A similar rise in available ETO_2_/FIO_2_concentrations was observed with the non-respiring mannequin, as depicted in Fig. 2. The addition of the overlying surgical mask did increase the available oxygen concentrations with the mannequin setup, however once the surgical mask was in place, there was no apparent additional increase in concentrations with an increase in O_2_ flow rates. The oxygen concentrations recorded are presented in Fig. 2.

### Nasal cannula vs simple oxygen mask (Hudson mask)

**Fig. 3:**
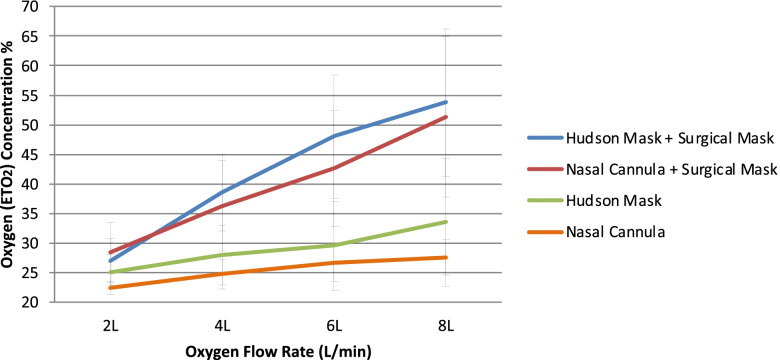
Comparison of measured mean ETO_2_ for all modalities studied with standard deviation bars

At all flow rates except one (nasal cannula + surgical mask, 2 L/min), the Hudson mask recorded higher mean ETO_2_ concentrations than the nasal cannula, as depicted in Fig. 3. The addition of a surgical facemask increased the mean ETO_2_ concentration from both the nasal cannula and the Hudson mask, with the nasal cannula + surgical mask outperforming the Hudson mask alone.

## Discussion

Surgical masks over oxygen devices are now commonplace. How does this alter the O_2_ concentration? We have found that at the start of inspiration this is enhanced in both devices. For nasal cannula at an O_2_ flow rate of 4 L/min the ETO_2_ is increased from 24.8 to 36.3% - improving the performance of the device by greater than one third. Thus, the nasal cannula with an overlying surgical mask would appear to “outperform” the unmodified Hudson mask. With the Hudson mask, the ETO_2_ also undergoes a similar enhancement, for example from 33.6 to 53.8% at 8 L/min. This offers a potential therapeutic benefit when higher levels of inspired oxygen are required, such as might occur in a resuscitation setting where high flow oxygen delivery via Hudson mask may be the only available therapy in a spontaneously ventilating patient.

The mechanism which produces this enhancement is not entirely clear. Our measuring system has limitations, and whilst having a rapid enough response time for delivery of anaesthesia, it is only able to give intermittent “snapshots” of the oxygen concentration delivered along its sampling tube; (Purchasing the proprietary software and hardware to download the “continuous” output from the Aisys CS2, still only gives a sampling time interval of 5 s). For this reason, our study is not designed to detect the rapid changes which during individual inspirations, and we were obliged to choose our measuring points carefully and restrict our conclusions to comparing like with like. By limiting the wide-open entrance to the airway, the surgical mask may well be decreasing the “entrainment of air” at peak inspiratory flows. As a speculation, we think it is also likely that the surgical mask is acting as a baffle, disrupting and therefore limiting the dissipation of oxygen away from the point of sampling, creating what is in effect a “virtual reservoir”. Further investigations into this effect would be worthwhile.

A recent study published by Binks, Parkinson and Sabbouh demonstrated that putting a surgical mask underneath a Hudson mask resulted in a negligible difference between FIO_2_ compared to an overlying surgical mask [25]. This study used a single volunteer and did not include values with no mask. We did not study using the surgical mask under the oxygen device; the superimposing technique is currently recommended by the Australian Council of Emergency Medicine and recent guidelines [12, 26]. Also of note, recent work by Mejia-Terrazas and Lopez-Munoz has recommended that an N95 mask be placed underneath a Hudson Mask [27]. Further research to analyse all three variables of no mask, underlying mask and overlying mask, using both N95 and surgical masks, would therefore be warranted for a definitive answer and clearer clinical picture.

Our basic study is useful, but our volunteers are not patients. They were at rest and not at all breathless. Their respiratory patterns are quite different from the sick, dyspnoeic patients who require these oxygen devices. Respiratory pattern, and specifically high inspiratory flow rates, will affect the relative amounts of oxygen and air that is drawn into the lungs, i.e. the FIO_2_ can change during every breath and we cannot replicate the multitude of possible respiratory patterns. Future studies should explore whether the effects of a surgical mask are similar in patients with respiratory compromise in the absence of COVID-19 infection. This would improve our understanding of economic oxygen utilisation in patients affected with COVID-19 during this pandemic.

The value displayed as ETO_2_ is measured by the AiSYS when the concentration of CO_2_ is at a maximum. This would naturally then be followed by a pause, and then an inspiration. The inspiration will draw in a variable amount of air and cause the value of O_2_ concentration to drop. Thus, the minimum measurable value of oxygen occurs at some time *during* inspiration (usually at the beginning of inspiration) depending on respiratory pattern and is a much less reliable data point. So, we have predominantly presented ETO_2_ - oxygen concentrations which represent a *maximum* oxygen concentration available when inspiration is about to start. This is “generous” to the devices as the real additional oxygen delivered to the alveoli corresponds to a lower FIO_2_.

As outlined earlier, there are concerns regarding the use of higher oxygen flow rates generating aerosols with subsequent increased transmission of viral particles. The general clinical approach is to give the minimum flow rate required to provide the patient with an adequate oxygen saturation, and in this respect, there are now more therapeutic options. We are fortunate that our facility has plenty of oxygen devices (and oxygen), but this is not the case everywhere, and it may be that nasal cannula and simple oxygen masks could be used with a surgical mask (or similar baffle) and provide crucial economies in oxygen usage, even in patients not on droplet precautions.

## Conclusions

This study has demonstrated that superimposing a surgical facemask over nasal cannula or a Hudson mask does not impair the available oxygen concentration and in fact appears to increase the available oxygen often by about one third. This may be regarded as a device performance enhancement in many cases, and allow for lower flow rates and prolongation of oxygen supplies. However, there are rarer cases where this increased oxygen concentration may result in diminished ventilation and CO_2_ retention and be deleterious to a patient.

While more work in this area is essential, this small study assists clinicians with an initial framework for oxygen prescription when overlying surgical masks are employed. Not only is this applicable in considering COVID-19 and other virus transmissions, but also where oxygen supplies are critically low and small savings in usage can be utilised to treat more patients.

## Supplementary Information

**Additional file 1: Figure 4.** Demonstration of oxygen sampling tube attached below the subject’s nares, with either nasal cannula (4a) or Hudson Mask (4b) overlying. **Figure 5.** Demonstration of placement of overlying surgical mask, with either nasal cannula (5a) or Hudson Mask (5b) underneath.

## Data Availability

The datasets used and analysed during the current study are available from the corresponding author on reasonable request.

## Abbreviations

CO_2_: Carbon dioxide
ETO_2_: End-tidal oxygen
FIO_2_: Fraction of inspired oxygen
L/min: Litres per minute
O_2_: Oxygen
