# Supplementary material for: The application of a surgical face mask over different oxygen delivery devices; a crossover study of measured end-tidal oxygen concentrations
Source: BMC Anesthesiol. 2022 Mar 7;22:62. doi: 10.1186/s12871-022-01602-y (PMC8899454; doi:10.1186/s12871-022-01602-y)
Supplement: Supplementary file 1 — Additional file 1: Figure 4. Demonstration of oxygen sampling tube attached below the subject’s nares, with either nasal cannula (4a) or Hudson Mask (4b) overlying. Figure 5. Demonstration of placement of overlying surgical mask, with either nasal cannula (5a) or Hudson Mask (5b) underneath. [file 12871_2022_1602_MOESM1_ESM.docx]

**APPENDIX**


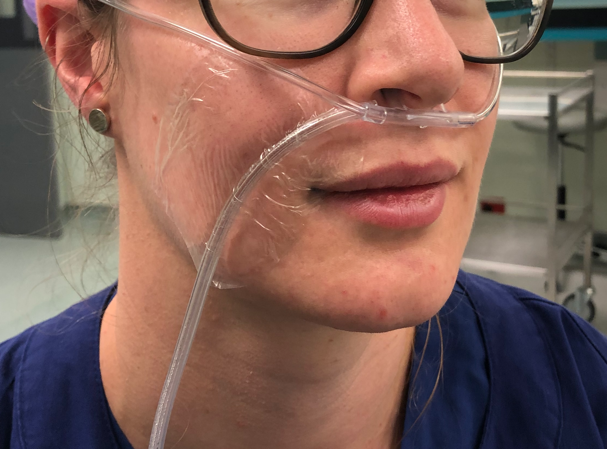

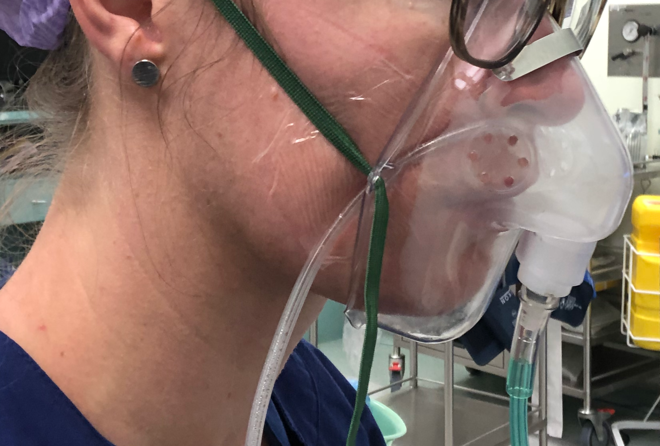


**4a 4b**

**Figure 4** Demonstration of oxygen sampling tube attached below the subject’s nares, with either nasal cannula (4a) or Hudson Mask (4b) overlying.


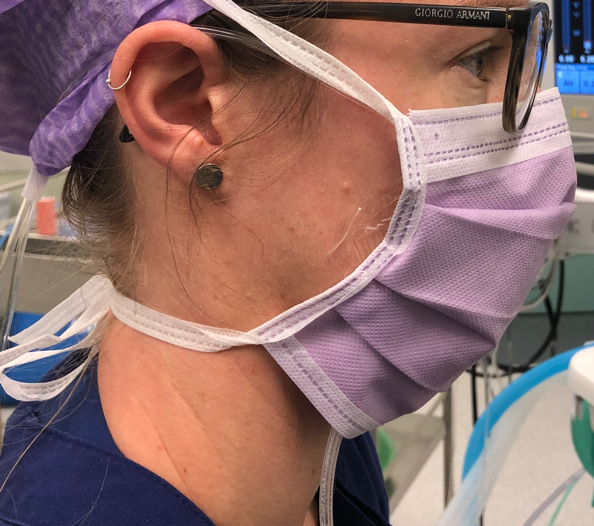

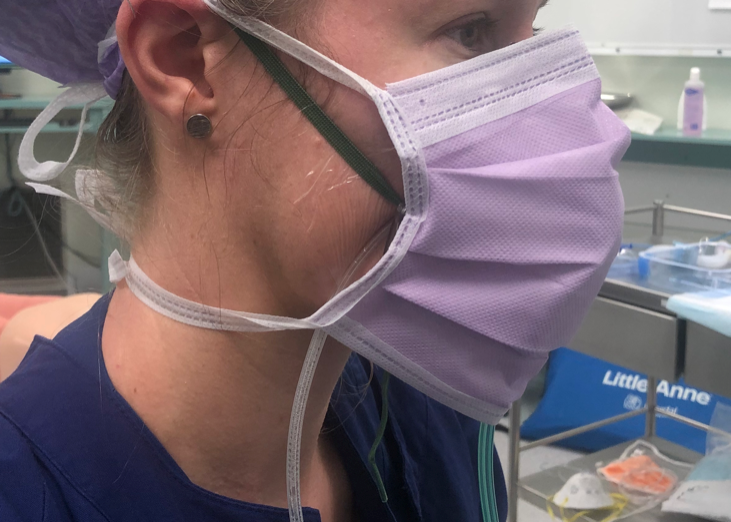


**5a 5b**

**Figure 5.** Demonstration of placement of overlying surgical mask, with either nasal cannula (5a) or Hudson Mask (5b) underneath.
